# Advanced rDNA-Based Detection of Wheat Pathogens in Grain Samples Using Next-Generation Sequencing (NGS)

**DOI:** 10.3390/pathogens14020164

**Published:** 2025-02-07

**Authors:** Katarzyna Pieczul, Ilona Świerczyńska, Andrzej Wójtowicz

**Affiliations:** Institute of Plant Protection—National Research Institute, 60-318 Poznan, Poland; k.pieczul@iorpib.poznan.pl (K.P.); i.swierczynska@iorpib.poznan.pl (I.Ś.)

**Keywords:** next-generation sequencing (NGS), ITS1 and ITS2, pathogen identification, fungi, wheat

## Abstract

High-throughput sequencing (HTS) has revolutionized phytopathology by overcoming many limitations of traditional diagnostic methods, as it permits precise pathogen monitoring, identification, and control, with ribosomal DNA (rDNA) regions serving as reliable markers for fungal classification. In this study, next-generation sequencing (NGS) was used, targeting the ITS1 and ITS2 regions to explore fungal diversity and pathogen presence in winter wheat grain samples and identifying 183 OTU sequences across 115 taxa. The ITS1 analysis yielded 249,743 reads, with *Fusarium* sp. (61%) as the dominant pathogenic taxon, followed by *Sporobolomyces* sp. (14%), *Cladosporium* sp. (3%), and other yeast-like or saprotrophic fungi, such as *Cryptoccocus* spp., *F. wieringae,* and *B. alba*. Sequencing of ITS1 also permitted the detection of *F. acuminatum* and the quarantine-regulated pathogens *T. caries* and *T. triticoides*. The ITS2 analysis produced 179,675 reads, with *F. culmorum* (47%) as the most abundant taxon, confirming significant grain contamination with this pathogen. Other frequently detected taxa included yeast-like fungi such as *C. tephrensis* (21%) and *V. victoriae* (13%), along with saprotrophic species like *S. roseus* and *Davidella* sp. ITS2 provided better resolution for the identification of *Fusarium* species by the detection of more pathogenic taxa associated with cereal diseases, including *F. culmorum,* as well as *F. cerealis*, *F. poae*, and *F. tricinctum*. The analysis revealed a diverse fungal community, including other pathogens such as *A. porri*, *B. cinerea*, and *C. herbarum*, as well as various non-pathogenic and saprotrophic fungal taxa. These findings underscore the complementary utility of ITS1 and ITS2 in profiling fungal diversity and detecting critical pathogens using HTS, highlighting the potential of these DNA regions for monitoring and managing cereal crop health.

## 1. Introduction

Identification of pathogenic fungi in agricultural products is crucial across many sectors. Given the importance of wheat (*Triticum aestivum* L.) as a staple crop in the global economy, the precise detection of grain contamination by pathogens is essential [1]. Such detections should permit the identification of the species that produce harmful mycotoxins and reduce grain quality, as well as regulate species subjected to phytosanitary controls [2]. Various methods are available to assess pathogen colonization in grains. They range from traditional mycological analyses that evaluate fungal growth on artificial media to grain sediment analysis [3]. Culturing fungi on synthetic media remains fundamental for visual assessment and preliminary pathogen identification through colony morphology and growth characteristics. While effective in some cases, these methods are labor-intensive, time-consuming, and restricted to taxa that can grow on artificial media, often favoring fast-growing species [4,5,6,7]. As a result, they are unreliable for the identification of obligate pathogens such as *Blumeria*, *Puccinia*, and *Tilletia*, which are the primary ones in cereal crops [8]. Furthermore, many pathogens may exhibit high intraspecific variability or are challenging to identify rapidly based solely on morphological criteria [9].

Besides offering certain unquestionable advantages, traditional methods are charged with limitations concerning diagnostic precision, sensitivity, and scalability [10]. To overcome these limitations, molecular techniques have been proposed as essential tools in pathogen detection. Methods like Enzyme-Linked Immunosorbent Assay (ELISA), PCR and its quantitative variant qPCR, Nested-PCR, Real-time PCR, Loop-Mediated Isothermal Amplification (LAMP), droplet digital PCR (ddPCR), Recombinase Polymerase Amplification (PRA), and other molecular techniques have provided accurate, rapid results for specific pathogens [4,10,11,12,13,14,15,16,17,18,19,20,21,22]. However, these techniques rely on primers or antibodies for each target, limiting detection to preselected species and potentially overlooking emerging or unexpected pathogens [15,16,23,24].

High-throughput sequencing (HTS) has significantly advanced the field of phytopathology, offering a diagnostic approach that bypasses many of the limitations of earlier methods [25]. The development of HTS has brought a transformative change to pathogen monitoring, identification, and the control of plant diseases [26]. Unlike traditional diagnostic methods that focus on the isolation and identification of individual species, HTS allows a comprehensive metagenomic analysis, revealing the diversity and structure of microbial populations within grain samples, including both culturable and non-culturable pathogens [13,25,27,28,29,30,31]. Furthermore, HTS has been applied in agricultural fields such as food safety, the identification of microorganism species in soil, the assessment of microbial community diversity and functionality, and the monitoring of fungicide resistance, among others [15,18,29,32,33,34,35]. HTS analyses can be valuable for the assessment of potential grain contamination by mycotoxins, permitting the identification of species under phytosanitary surveillance, and detecting beneficial organisms that may support plant health or compete with pathogenic fungi. Yet, HTS is especially valuable for studying obligate fungi, which cannot grow on synthetic media. This capacity is particularly precious for studying cereal crop pathogens, such as those from the genera *Blumeria*, *Puccinia*, and *Tilletia* [36,37].

For fungal species identification and classification, ribosomal DNA (rDNA) regions are among the most reliable and widely used genetic markers [16,24,38,39]. The ITS region comprises partitions—ITS1, 5.8S, and ITS2, with the ITS regions exhibiting high variability among different fungal species [40]. Within rDNA, the internal transcribed spacer (ITS) regions are recognized as the primary markers for fungal taxonomy and identification [38,39,40]. The high variability of ITS regions makes them ideal for the differentiation of fungal species. ITS regions have become the standard for the distinction of even closely related fungal taxa [39,41]. To enhance specificity in the identification of fungal DNA, fungi-specific ITS primers that selectively amplify fungal ITS regions while minimizing interference from non-target DNA, such as plant or bacterial DNA, have been developed. This specificity is crucial for the precise characterization of fungal communities in environmental and agricultural samples, where fungi are often present alongside other organisms [25,40].

The aim of this study was to investigate the suitability of ITS1 vs. ITS2 sequence clusters for the detection of wheat grain pathogens to optimize pathogen identification in agricultural contexts. The findings highlight the advantages of dual-region sequencing for comprehensive pathogen identification, suggesting that a customized approach tailored to each study type can provide the most accurate and reliable insights. These applications underscore the value of ITS-based identification for advancing knowledge in mycology and improving the management of fungi harmful to crop and food security.

## 2. Materials and Methods

### 2.1. Plant Material

The winter wheat grain (*Triticum aestivum* L.) variety Euforia was collected in August 2022 from the Winna Góra Experimental Station of the Plant Protection Institute—National Research Institute in Poland (52.1873° N, 17.2953° E). Three independent 500 g samples of untreated grain (not exposed to fungicide applications) were collected from three plots and combined to form a composite sample. The resulting stock batch was stored at room temperature for three months before further analyses were conducted.

### 2.2. DNA Isolation

Fifty grams of grain from the composite sample was weighed and placed into a container. Next, 100 mL of distilled water was added to the sample, and the mixture was manually shaken for 10 min to ensure thorough contact. After shaking, the grains were removed and discarded, while the resulting liquid was transferred to sterile 50 mL Falcon tubes. The tubes were centrifuged at 6000 rpm (equivalent to 4600× *g*) for 5 min using an Eppendorf 5804 centrifuge. The resulting pellet was gently resuspended for total DNA extraction using the Plant/Fungi DNA Isolation Kit (Norgen Biotek, Thorold, ON, Canada). For each trial, 30 mg of the pellet was processed according to the manufacturer’s instructions for DNA isolation. The concentration of the isolated DNA was then measured using a NanoDrop spectrophotometer (Thermo Fisher Scientific, Waltham, MA, USA) to ensure accurate quantification of the DNA yield.

### 2.3. PCR Reaction

Based on literature recommendations for amplification of the ITS1 and ITS2 regions, locus-specific primers ITS1-F-KYO2 (5′TAGAGGAAGTAAAAGTCGTAA3′) and ITS2-KYO2 (5′TTYRCTRCGTTCTTCATC3′), as well as ITS3-KYO2 (5′GATGAAGAACGYAGYRAA3′) and ITS4-F-KYO2 (5′RBTTTCTTTTCCTCCGCT3′) [40], each containing a 5′ overhang for indexing, were used. The PCR mixture contained 1 μL of template DNA (30 ng/μL), 1 μM of each forward and reverse primer, 200 μM dNTPs (Thermo Fisher Scientific), 1× HF Buffer (Thermo Fisher Scientific), and 0.2 U Hot Start Phusion polymerase (Thermo Fisher Scientific). Separate PCR reactions for the ITS1 and ITS2 regions were conducted at three different annealing temperatures in a total volume of 20 μL. The thermal cycling protocol was as follows: initial denaturation at 95 °C for 3 min, followed by 40 cycles of denaturation at 95 °C for 30 s; annealing at 50 °C, 53 °C, and 56 °C for 30 s at each temperature; and elongation at 72 °C for 30 s, with a final extension step at 72 °C for 5 min. All PCR reactions were carried out using a Mastercycler ep gradient S thermal cycler (Eppendorf, Hamburg, Germany). Then, 5 μL of the amplified PCR products from each sample (50, 53, and 56 °C) were used as the templates for NGS library preparation. The pooled samples were quantified using the Quant-iT PicoGreen dsDNA Assay Kit (Thermo Fisher Scientific).

### 2.4. NGS Library Preparation, Sequencing, and Bioinformatics Analysis

Based on the provided PCR amplicons, NGS library preparation, sequencing, and bioinformatics analysis were performed by a commercial service—Genomed SA (Warsaw, Poland). The PCR amplicons were purified using Agencourt AMPure XP beads (Beckman Coulter, Brea, CA, USA) and quantified with the Quant-iT PicoGreen dsDNA Assay Kit (Thermo Fisher Scientific). Indexing was carried out using the Nextera XT Dual-Index Kit (Illumina, San Diego, CA, USA) according to the manufacturer’s instructions. Sequencing was subsequently performed in 2 × 250 bp paired-end mode on the Illumina MiSeq platform. Primary analysis, including base calling and demultiplexing, was automatically performed on the MiSeq system using MiSeq Reporter (version 2.6). Adapter and quality trimming were conducted using Cutadapt (version 1.9.1) with parameters set to a minimum read length of 30 bp and a minimum base quality of 20. The paired-end reads (Read 1 and Read 2) were joined using the fastq-join algorithm within the QIIME package. Chimeric sequences were filtered out using usearch61 before the clustering step. The UCLUST algorithm, clustering at 97% sequence similarity, combined with the BLAST algorithm, was used to assign taxonomy to the OTU (Operational Taxonomic Unit) representative sequences. Taxonomic assignment of the OTU representative sequences was carried out using the BLAST algorithm against NCBI’s nt database. The obtained data were deposited in the Sequence Read Archive (SRA) under BioProject PRJNA1219055.

## 3. Results

**Figure 1:**
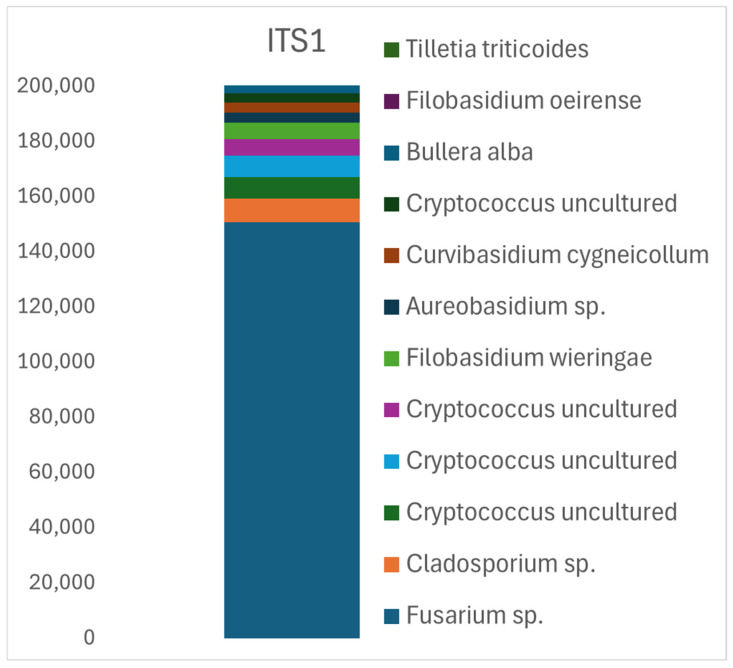
Relative abundance of fungal taxa identified in ITS1 sequence analysis of the 12 most common OTUs. The numerical scale on the left corresponds to the number of sequences read for each OTU.

The ITS1 analysis yielded 249,743 quality-filtered reads, identifying 75 representative OTU sequences corresponding to 56 distinct taxa at the species or genus level. The most frequently detected species was *Fusarium*, accounting for 61% of the reads (150,690). Other commonly identified taxa included *Sporobolomyces* sp. (14%, 34,660 reads) and four distinct strains of *Cryptococcus*, each representing separate OTUs and collectively contributing 3.2–1.4% of the reads (7842; 7778; 5919; and 3496). These strains, typically yeast-like and often found in many ecosystems, reflect the diversity of fungal communities in natural habitats. *Cladosporium* sp., one of the most widespread and diverse fungal genera, accounted for 3% of the reads (8550). Additional yeast-like or saprotrophic fungi included *Filobasidium wieringae*—2.3% and species like *Aureobasidium* sp., *Curvibasidium cygneicollum*, *Sporobolomyces* sp., and *B. alba* represented 1.6–1.2% of the reads, showcasing the ecological diversity captured by the ITS1 marker (Table 1, Figure 1).

The ITS1 analysis did not provide sufficient resolution to differentiate individual taxa within the *Fusarium* genus except for *Fusarium acuminatum* (263 reads) and a single read of *Fusarium proliferatum*. Despite this limitation, ITS1 permitted the detection of 205 sequence reads of quarantine-regulated pathogens *Tilletia caries* and 1272 reads of *Tilletia triticoides* (Table 1). Another major pathogen identified in only 14 reads was *Zymoseptoria tritici*, the causative agent of Septoria leaf blotch, a destructive disease of wheat that can significantly reduce yield. Given its impact, *Z. tritici* is monitored in multiple countries to manage its spread and significant crop losses. These results highlight the ITS1 region’s capacity to detect ecologically and economically relevant fungal taxa despite certain limitations in taxonomic resolution.

**Figure 2:**
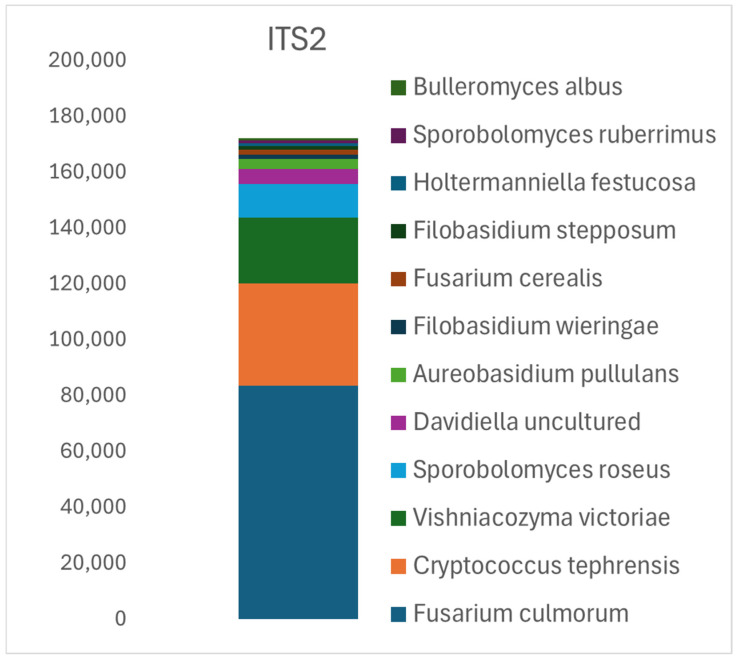
Relative abundance of fungal taxa identified in ITS2 sequence analysis of the 12 most common OTUs. The numerical scale on the left corresponds to the number of sequences read for each OTU.

The ITS2 analysis produced 179,675 quality-filtered reads, identifying 108 representative OTU sequences corresponding to 71 taxa at the species or genus level. The most frequently detected species was *Fusarium culmorum*, which accounted for 47% of all reads (83,554), suggesting a significant level of grain contamination with this pathogen. Other prevalent species included yeast-like fungi such as *Cryptococcus tephrensis* (21%, 36,582 reads), *Vishniacozyma victoriae* (13%, 23,544 reads), and *Sporobolomyces roseus* (7%, 11,973 reads), as well as the saprotrophic species *Davidella* sp. (3%, 5580 reads) (Figure 2). Unlike ITS1, the ITS2 analysis enabled the identification of multiple distinct *Fusarium* species. In addition to the dominant *F. culmorum*, the study detected 1735 reads of *Fusarium cerealis* sequences, 56 reads of *Fusarium poae*, 12 reads of *Fusarium langsethiae* (NR121214), 3 of *Fusarium tricinctum*, and 2 of *Fusarium brasilicum* (Table 1, Figure 2). These results highlight the utility of ITS2 for distinguishing closely related *Fusarium* taxa, which are significant pathogens in cereal crops that cause diseases such as Fusarium head blight and root rot. These conditions can lead to substantial reductions in both crop yield and grain quality. However, certain key pathogens, including *T. caries*, *T. triticoides*, and *Z. tritici*, were effectively identified only through ITS1 analysis (Table 1). This suggests that a combined approach using both ITS1 and ITS2 may provide a more comprehensive assessment of fungal diversity and pathogen presence.

We observed notable differences in taxonomic assignments between ITS1 and ITS2 regions at both the genus and species levels. ITS2 analysis identified more taxa and facilitated more precise differentiation of *Fusarium* species compared to ITS1. Summarizing a combined analysis of the ITS1 and ITS2 regions permitted the identification of 183 representative OTU sequences corresponding to 115 distinct taxa. This approach also revealed additional potentially pathogenic species not previously mentioned, such as *Alternaria porri*, *Ascochyta hordei*, *Aureobasidium pullulans*, *Botrytis cinerea*, *Cladosporium herbarum*, *Epicoccum pimprinum*, and *Ulocladium dauci* (Table 1) and representatives from the genera *Microdochium*, *Phaeosphaeria*, and *Stemphylium*. Many identified fungal species alongside various yeast species were weak pathogens or saprotrophs. The most frequently detected non-pathogenic species included *C. tephrensis*, *V. victoriae*, *S. roseus*, *F. wieringae*, *Davidiella* sp., *Aureobasidium* spp., *B. alba*, and *Rhodotorula* sp. (Table 1). Interestingly, a significant diversity was observed within the genus *Cryptococcus*, with 12 distinct OTUs identified using ITS1 (10% of reads) and 29 OTUs using ITS2 (21% of reads). While these species are generally non-pathogenic to cereals, they are commonly found on crops and in soil, contributing to the broader microbial ecosystem and indicating the importance of these fungi in shaping microbial diversity in agricultural systems.

## 4. Discussion

The development of molecular biology techniques leveraging HTS has revolutionized phytopathological research. HTS facilitates the comprehensive analysis of entire fungal communities through universal primers, eliminating the need for the prior selection of target species [30,40,41]. This innovative approach allows for the detection of diverse organisms, including unexpected or novel pathogens, that might escape identification using traditional or targeted molecular methods. By enabling the broad analysis of microbial communities, HTS can effectively address numerous challenges in the field. While this approach is precious for many studies, the wide range of data can complicate pathogen-focused research. The unintended detection of environmental or saprotrophic fungi adds complexity, mainly when the study is aimed at the analysis of pathogenic species exclusively. HTS analyses also do not allow the determination of the degree of pathogenicity of the identified pathogenic species toward host plants. It is necessary to use additional bioinformatic processing and robust computational tools to distinguish between pathogenic and non-pathogenic species.

In our analysis, many reads were classified as concerning non-pathogenic or saprotrophic species. For the ITS1 region, approximately 35% of the identified OTUs were assigned to non-pathogenic taxa, including *Sporobolomycetes*, *Cryptococcus*, *Filobasidium*, *Aureobasidium*, *Curvibasidium*, and *Dioszegia* (Table 1). In the case of the ITS2 region, the proportion of OTUs classified as non-pathogenic taxa was around 50% of the ITS2 reads. The dominant taxa for this region included *Cryptococcus*, *Vishniacozyma*, *Sporobolomycetes*, *Davidiella*, *Aureobasidium*, *Filobasidium*, *Holtermanniella*, and members of the *Dikarya* clade (Table 1). Certain fungi play beneficial roles in agricultural systems by supporting crop health and inhibiting pathogens through competitive exclusion or the secretion of antifungal compounds [42]. These non-pathogenic reads occupy a significant proportion of OTUs, which can reduce pathogen detectability. Similar studies highlight the challenges of distinguishing pathogenic from non-pathogenic species within fungal communities. Nicolaisen [30] also observed that non-pathogenic or opportunistic fungal species often dominate HTS analyses of crop-associated communities. It emphasizes the need for more refined analysis techniques in pathogen-focused research. To improve specificity, primers that target specific taxonomic groups or pathogen-related sequences could be applied to prioritize pathogen detection in HTS analyses [43]. Piombo [44] has emphasized the potential of high-throughput sequencing (HTS) techniques as powerful tools for monitoring soilborne, seaborne, and airborne pathogens. These methods are also promising for the identification of novel pathogens and tracing the origins of outbreaks. However, their effectiveness relies on the careful selection of primers and bioinformatic algorithms. Additionally, advancements in bioinformatics are expected to significantly accelerate the adoption of metagenomics for the detection and monitoring of plant pathogens [44]. Long-read HTS methods, such as SMRT and nanopore sequencing, allow complete sequencing of the ITS region (ITS1-5.8S-ITS2) in a single read, reducing ambiguities from fragmentation. While offering significant advantages, these methods are more expensive, require higher-quality DNA, and present bioinformatic challenges, including higher error rates and reliance on incomplete or biased databases [45,46].

Numerous authors have extensively investigated the selection of ITS primers and the optimization of PCR reaction parameters to enable the analysis of the broadest possible spectrum of fungi. However, the debate over whether ITS1 or ITS2 offers better taxonomic resolution remains unresolved [47]. Some studies have shown that ITS1 is generally more variable than ITS2 across many fungal taxons [48,49,50,51]. However, Bazzicalupo [48] has also demonstrated that ITS2 may exhibit more significant variability and provide a more accurate reflection of the molecular diversity of environmental samples. Other authors have concluded that ITS1 and ITS2 are suitable markers for DNA metabarcoding and yield comparable results [47,52]. Yang [53] has also indicated that analyses using ITS1 and ITS2 revealed distinct taxa sets but similar taxonomic preferences. It has also been suggested that ITS2 could be an effective marker for assessing operational taxonomic richness and taxonomic details of fungal communities, mainly when the full-length ITS sequence is unavailable. In the studies conducted by Mbareche [50], some taxa were detected using ITS2, but some other ones were detected using ITS1 only. However, the shotgun metagenomic approach showed a taxonomic profile more similar to ITS1 than ITS2 [50].

Our results confirm those provided by Scibetta et al. [54], who, in a comparative study, observed that primer sets targeting the ITS1 region proved to be more specific than those targeting the ITS2 region. However, according to these authors, a perfect set of primers to investigate the whole fungal diversity does not exist. Whatever the choice, only a fraction of the actual microbial diversity will be explored: the primers have to be selected according to the objective of the analysis so that reliable fungal identification should incorporate sequencing data from both the ITS1 and ITS2 regions. Most of the identified species were detected using either ITS1 or ITS2 individually, with only a limited number identified across both regions. The latter included *Fusarium* spp., *B. cinerea*, *C. tephrensis*, *Dioszegia crocea*, *Filobasidium oeirense*, *Hannaella coprosmae*, *Sporobolomyces ruberrimus*, *Udeniomyces pyricola*, *V. victoriae*, and others (Table 1). These findings suggest that the range of identified species differs depending on the use of ITS1 or ITS2 in standard analyses. A combination of both markers provides a more comprehensive assessment of population complexity. The importance of this issue is expected to diminish with advancements in HTS techniques, enabling the precise analysis of larger DNA fragments spanning ITS1 and ITS2. In the study, more taxa were identified using ITS2 compared to ITS1, highlighting ITS2’s potentially broader utility for certain fungal groups (Table 1). However, the selection of the appropriate marker region should be made considering factors such as amplification efficiency, variability, and compatibility with reference databases to optimize detection and identification. The ITS1 region permitted the effective identification of critical pathogens such as *T. caries*, *T. tricinctum*, and *Z. tritici*. Species from the genus *Tilletia* are under monitoring in many countries, including the United States, Australia, and various EU ones, due to their potential to cause significant yield losses in wheat crops. On the other hand, the ITS2 region enabled the more precise identification of *Fusarium* species and facilitated the detection of additional genera, such as *Alternaria*, *Ascochyta*, *Cladosporium*, *Epicoccum*, *Rhodotorula*, *Sporobolomyces*, and *Vishniacozyma*. This broader target offers a more comprehensive view of fungal diversity (Table 1).

Apart from the differences mentioned above, the dominant genus in the ITS1 and ITS2 regions was *Fusarium*. In our study, *Fusarium* species dominated the pathogenic fungal sequence readings. For the ITS1 region, 61% of the reads corresponded to *Fusarium* sp. In the ITS2 analysis, the results were even more specific, with 47% of the most common reads attributed to *F. culmorum*, indicating significant contamination of the examined grain by this species (Figure 1 and Figure 2). This is crucial information, as in Central Europe, *F. culmorum*, *F. graminearum*, and *F. sporotrichioides* are the primary fungal pathogens affecting cereal grains. All these species produce harmful trichothecene mycotoxins, such as deoxynivalenol (DON) and nivalenol (NIV), which persist through food processing and pose significant health risks [55,56,57].

For a large number of reads, the presence of a particular pathogen species is unequivocal. However, a considerable portion of results pertains to single or very few reads within an OTU. When identifying key pathogens, an unresolved question remains: what is the minimum number of reads necessary to confirm the presence of a species in the analyzed sample?

The use of both markers offers a broader perspective on the fungal population. However, each marker provides varying levels of precision, depending on the fungal taxa present. The analysis of both ITS regions enhances the reliability of results, although this approach increases analysis costs, which is a notable drawback. HTS requires access to well-equipped laboratories, skilled personnel, and complex, time-consuming bioinformatics analyses.

Additionally, it relies on robust bioinformatics tools and reliable reference databases, which are often limited. Another limitation is that HTS analysis does not provide quantitative data on grain contamination levels. Furthermore, the DNA isolation process can significantly impact the outcomes, as the efficiency of DNA extraction varies between species. Additionally, the choice of primers plays a pivotal role in shaping which fungal species are detected in the samples [25,51].

To summarize, HTS analysis has emerged as a highly effective tool for the accurate detection and identification of pathogens. The data obtained offer valuable insights into the complete structure of organisms colonizing grain, revealing potential contamination by mycotoxin-producing species and aiding in the identification of regulated pathogens subject to monitoring and control measures. Despite its many advantages, HTS has limitations that warrant careful consideration when interpreting results. A combination of HTS with complementary methods, such as PCR and traditional culturing, offers a more precise diagnostic approach for phytopathology, improving pathogen detection and overall diagnostic reliability. We can expect that in the near future, long-read technologies will become a valuable tool in fungal research, particularly in resolving complex or highly variable ITS regions. As costs decrease and tools advance, these technologies have the potential to significantly support fungal taxonomy, phylogenetics, and environmental studies.

## Figures and Tables

**Table 1:** Taxonomic classification of dominant fungal species identified using the NCBI Database: number and percentage of sequences derived from ITS1 and ITS2 regions.

NCBI Reference Ac. Nr.	Taxonomic Classification			Number and Percentage of Sequencing Reads			
	Family	Genus	Species	ITS1		ITS2	
JQ818176	*Pleosporaceae*	*Alternaria*	*Alternaria porri*	0	0.0	19	0.0
HQ882800	*Didymellaceae*	*Ascochyta*	*Ascochyta hordei*	0	0.0	2	0.0
KX869960	*Aureobasidiaceae*	*Aureobasidium*	*Aureobasidium pullulans*	0	0.0	3393	1.9
KY305025	*Aureobasidiaceae*	*Aureobasidium*	*Aureobasidium* sp.	3878	1.6	0	0.0
KY319171	*Sclerotiniaceae*	*Botrytis*	*Botrytis cinerea*	21	0.0	10	0.0
KX096691	*Tremellaceae*	*Buckleyzyma*	*Buckleyzyma aurantiaca*	0	0.0	13	0.0
KY101819	*Bulleraceae*	*Bullera*	*Bullera alba*	3009	1.2	0	0.0
KX096662	*Bulleraceae*	*Bulleromyces*	*Bulleromyces albus*	0	0.0	822	0.5
JN906979	*Cladosporiaceae*	*Cladosporium*	*Cladosporium arthropodii*	0	0.0	41	0.0
JF770450	*Cladosporiaceae*	*Cladosporium*	*Cladosporium grevilleae*	0	0.0	92	0.1
EF679363	*Cladosporiaceae*	*Cladosporium*	*Cladosporium herbarum*	0	0.0	13	0.0
KY305063	*Cladosporiaceae*	*Cladosporium*	*Cladosporium* sp.	8550	3.4	0	0.0
KX674650	*Cladosporiaceae*	*Cladosporium*	*Cladosporium tenellum*	0	0.0	770	0.4
KX096664	*Tremellaceae*	*Cryptococcus*	*Cryptococcus carnescens*	0	0.0	106	0.1
AY188365	*Tremellaceae*	*Cryptococcus*	*Cryptococcus dimennae*	0	0.0	14	0.0
AB085806	*Tremellaceae*	*Cryptococcus*	*Cryptococcus flavescens*	0	0.0	51	0.0
KM408426	*Tremellaceae*	*Cryptococcus*	*Cryptococcus foliicola*	0	0.0	14	0.0
LK023834	*Tremellaceae*	*Cryptococcus*	*Cryptococcus frias*	0	0.0	154	0.1
JQ247574	*Tremellaceae*	*Cryptococcus*	*Cryptococcus laurentii*	0	0.0	12	0.0
EF126366	*Tremellaceae*	*Cryptococcus*	*Cryptococcus magnus*	0	0.0	38	0.0
KX096667	*Tremellaceae*	*Cryptococcus*	*Cryptococcus tephrensis*	0	0.0	36,582	20.7
AM160647	*Tremellaceae*	*Cryptococcus*	*Cryptococcus tephrensis*	0	0.0	180	0.1
HG935846	*Tremellaceae*	*Cryptococcus*	*Cryptococcus* uncultured	0	0.0	801	0.5
KY430484	*Tremellaceae*	*Cryptococcus*	*Cryptococcus* uncultured	7842	3.2	0	0.0
KY430574	*Tremellaceae*	*Cryptococcus*	*Cryptococcus* uncultured	7778	3.1	0	0.0
KY439562	*Tremellaceae*	*Cryptococcus*	*Cryptococcus* uncultured	5919	2.4	0	0.0
KY430500	*Tremellaceae*	*Cryptococcus*	*Cryptococcus* uncultured	3496	1.4	0	0.0
KY102971	*Curvibasidiaceae*	*Curvibasidium*	*Curvibasidium cygneicollum*	3533	1.4	471	0.3
KY305024	*Cystofilobasidiaceae*	*Cystofilobasidium*	*Cystofilobasidium capitatum*	224	0.1	0	0.0
KY103168	*Cystofilobasidiaceae*	*Cystofilobasidium*	*Cystofilobasidium infirmominiatum*	39	0.0	0	0.0
KY103181	*Cystofilobasidiaceae*	*Cystofilobasidium*	*Cystofilobasidium macerans*	528	0.2	2	0.0
KY430536	*Cystofilobasidiaceae*	*Cystofilobasidium*	*Cystofilobasidium* uncultured	966	0.4	0	0.0
HG935280	*Mycosphaerellaceae*	*Davidiella*	*Davidiella* uncultured	0	0.0	5580	3.2
KY070283	*Didymellaceae*	*Didymella*	*Didymella americana*	24	0.0	0	0.0
KY103351	*Tremellaceae*	*Dioszegia*	*Dioszegia crocea*	1210	0.5	7	0.0
KX096671	*Tremellaceae*	*Dioszegia*	*Dioszegia fristingensis*	0	0.0	13	0.0
KX067808	*Tremellaceae*	*Dioszegia*	*Dioszegia hungarica*	0	0.0	232	0.1
KY103358	*Tremellaceae*	*Dioszegia*	*Dioszegia rishiriensis*	27	0.0	10	0.0
KY430571	*Tremellaceae*	*Dioszegia*	*Dioszegia* uncultured	971	0.4	0	0.0
AF444354	*Filobasidiaceae*	*Filobasidium*	*Filobasidium chernovii*	0	0.0	179	0.1
KY103438	*Filobasidiaceae*	*Filobasidium*	*Filobasidium oeirense*	1578	0.6	627	0.4
KX067805	*Filobasidiaceae*	*Filobasidium*	*Filobasidium stepposum*	0	0.0	1232	0.7
KY103450	*Filobasidiaceae*	*Filobasidium*	*Filobasidium wieringae*	5818	2.3	1746	1.0
KX869940	*Nectriaceae*	*Fusarium*	*Fusarium acuminatum*	263	0.1	0	0.0
DQ459861	*Nectriaceae*	*Fusarium*	*Fusarium brasilicum*	0	0.0	2	0.0
KF889097	*Nectriaceae*	*Fusarium*	*Fusarium cerealis*	0	0.0	1735	1.0
LT598662	*Nectriaceae*	*Fusarium*	*Fusarium culmorum*	0	0.0	83,554	47.2
NR121214	*Nectriaceae*	*Fusarium*	*Fusarium langsethiae*	0	0.0	12	0.0
KC311480	*Nectriaceae*	*Fusarium*	*Fusarium poae*	0	0.0	56	0.0
MF076579	*Nectriaceae*	*Fusarium*	*Fusarium* sp.	150,690	60.8	0	0.0
HM776425	*Nectriaceae*	*Fusarium*	*Fusarium tricinctum*	0	0.0	3	0.0
KY103497	*Hannaellaceae*	*Hannaella*	*Hannaella coprosmae*	93	0.0	55	0.0
MF125294	*Tremellaceae*	*Holtermanniella*	*Holtermanniella festucosa*	0	0.0	1131	0.6
KY558354	*Tremellaceae*	*Kwoniella*	*Kwoniella pini*	19	0.0	0	0.0
KY104319	*Tremellaceae*	*Naganishia*	*Naganishia albidosimilis*	29	0.0	0	0.0
KY104695	*Sporidiobolaceae*	*Rhodosporidiobolus*	*Rhodosporidiobolus colostri*	172	0.1	41	0.0
FN812728	*Sporidiobolaceae*	*Rhodotorula*	*Rhodotorula* uncultured	281	0.1	0	0.0
KX067834	*Sporidiobolaceae*	*Sporobolomyces*	*Sporobolomyces roseus*	48	0.0	11,973	6.8
KX067835	*Sporidiobolaceae*	*Sporobolomyces*	*Sporobolomyces ruberrimus*	54	0.0	829	0.5
KC753421	*Sporidiobolaceae*	*Sporobolomyces*	*Sporobolomyces* uncultured	0	0.0	639	0.4
KT334689	*Sporidiobolaceae*	*Sporobolomyces*	*Sporobolomyces* uncultured	34,660	14.0	0	0.0
MF039598	*Sporidiobolaceae*	*Sporobolomyces*	*Sporobolomyces* uncultured	3153	1.3	0	0.0
AF398436	*Tilletiaceae*	*Tilletia*	*Tilletia caries*	205	0.1	0	0.0
AF398446	*Tilletiaceae*	*Tilletia*	*Tilletia triticoides*	1272	0.5	0	0.0
KY109991	*Sporidiobolaceae*	*Udeniomyces*	*Udeniomyces pyricola*	311	0.1	51	0.0
KY075674	*Pleosporaceae*	*Ulocladium*	*Ulocladium dauci*	40	0.0	0	0.0
KY105819	*Filobasidiaceae*	*Vishniacozyma*	*Vishniacozyma carnescens*	350	0.1	0	0.0
KY105823	*Filobasidiaceae*	*Vishniacozyma*	*Vishniacozyma globospora*	24	0.0	0	0.0
KY430573	*Filobasidiaceae*	*Vishniacozyma*	*Vishniacozyma* uncultured	942	0.4	0	0.0
LC203739	*Filobasidiaceae*	*Vishniacozyma*	*Vishniacozyma victoriae*	5	0.0	23,544	13.3
LT882682	*Mycosphaerellaceae*	*Zymoseptoria*	*Zymoseptoria tritici*	14	0.0	0	0.0
	The remaining taxa not included in the analysis			1707	0.7	2824	1.6
The total number of reads				249,743		179,675	

## Data Availability

The data supporting the results of this study are available from the author (K.P.) upon reasonable request.
